# News and Notes

**Published:** 1905-11

**Authors:** 


### NEWS AND NOTES.

THE first of the German sanatoriums founded on the Island of Madeira by Prince Hohenlohe is now opened.

IT is announced that Prof. von Jaksch, of Prague, has been called to Vienna to take Dr. Nothnagels' chair.

THE lord mayor of Leeds, England, has determined to start a system of pure milk depots in that town to check the fearful infantile mortality there.

INSTRUCTION in First Aid to the Injured is to be given to the New York police force by Dr. Kilmer, who has been specially engaged for this service.

THE Marine-Hospital Service has issued a warning against lobsters, claiming that they are aiding the spread of typhoid, now so prevalent in many Eastern cities.

THE disagreeable odor of ichthyol, a drug too valuable to be neglected on this account, can be concealed by the addition of oil of citronella, 20 minims to the ounce of ointment.—*Ex.*

GENERAL LEONARD WOOD, the sanitary reformer of Cuba, has lately been operated on successfully for an exostosis of the skull, the result of a blow on the crown of the head received about a year ago.

THE meeting of the Louisiana State Board of Medical Examiners, which was to have been held on October 20th and 21st, has, on account of the present uncertain conditions of travel, been postponed.

Dr. Suzuki, Surgeon-General of the Royal Japanese Navy, is a visitor to America. He attended as a special guest of honor the recent meeting of the American Association of Obstetricians and Gynecologists in New York.

---

Professor Osler, of Oxford, has accepted the post of Thomas Young, Lecturer on Medicine at St. George's Hospital, and will give a series of lectures and demonstrations at the hospital next spring on the diagnosis of abdominal tumors.

---

On November 11th the medical profession of Chicago will tender a banquet to Dr. Nicholas Senn. The committee of arrangements is composed of the following men: Drs. Wm. A. Evans, Frank Billings, John B. Murphy, Wm. L. Baum and David J. Doherty.

---

Attention is called to an ad in this number placing before the profession a meritorious article, "Kefilac Tablets." Whoever knows the value of Kononyss and the difficulty of obtaining it, will be glad of the opportunity of getting a tablet, which enables the patient to make Kononyss or Kefilac at his home without any difficulty.

---

It has been announced by Dr. J. H. White, United States Marine Hospital Surgeon-in-charge in New Orleans, that the new mosquito exterminator known as "Culicide," discovered by Dr. Mims, chemist for the city Board of Health, will be adopted by the Government. Dr. White announced that the disinfectant is composed of equal parts of carbolic acid and gum-camphor. He has advised strongly against amateur use of the composition owing to its explosive character. None but skilled employees of the United States Marine Hospital Service will be permitted to use it.

